# Supplementary material for: Dynamic multi-species occupancy models reveal individualistic habitat preferences in a high-altitude grassland bird community
Source: PeerJ. 2019 Feb 15;7:e6276. doi: 10.7717/peerj.6276 (PMC6378914; doi:10.7717/peerj.6276)
Supplement: Appendix S1 — Scoring of prevailing weather conditions (observability) each weighted according to how DHM based on knowledge of the area perceived a weather variable to nfluence detection of birds. Conditions were optimal with a clear sky (score 1 = 100), cool emperatures (score 2 = 100) and calm wind conditions (score 3 = 100). For other weather conditions, observability was reduced and we chose the scores according to our subjective. [file peerj-07-6276-s001.docx]

| **score 1** | **score 2** | **score 3** | **sky** | **temp** | **wind** | **% observabilty** |
| --- | --- | --- | --- | --- | --- | --- |
| 100 | 100 | 100 | clear | cool | calm | 100 |
| 100 | 100 | 80 | clear | cool | moderate | 93.33 |
| 100 | 100 | 60 | clear | cold | strong | 86.67 |
| 100 | 50 | 80 | clear | cold | moderate | 76.67 |
| 100 | 50 | 60 | clear | cold | strong | 70 |
| 100 | 60 | 100 | clear | hot | calm | 86.67 |
| 100 | 60 | 80 | clear | hot | moderate | 80 |
| 100 | 60 | 60 | clear | hot | strong | 73.33 |
| 70 | 100 | 100 | cloudy | cool | calm | 90 |
| 70 | 50 | 80 | cloudy | cold | moderate | 66.67 |
| 70 | 50 | 60 | cloudy | cold | strong | 60 |
| 70 | 100 | 80 | cloudy | cool | moderate | 83.33 |
| 70 | 100 | 60 | cloudy | cool | strong | 76.67 |
| 70 | 70 | 100 | cloudy | warm | calm | 80 |
| 50 | 100 | 100 | misty | cool | calm | 83.33 |
| 50 | 100 | 80 | misty | cool | moderate | 76.67 |
| 50 | 100 | 60 | misty | cool | strong | 70 |
| 80 | 50 | 80 | partly cloudy | cold | moderate | 70 |
| 80 | 50 | 60 | partly cloudy | cold | strong | 63.33 |
| 80 | 100 | 100 | partly cloudy | cool | calm | 93.33 |
| 80 | 100 | 80 | partly cloudy | cool | moderate | 86.67 |
| 80 | 100 | 60 | partly cloudy | cool | strong | 80 |
| 80 | 60 | 100 | partly cloudy | hot | calm | 80 |
| 80 | 60 | 80 | partly cloudy | hot | moderate | 73.33 |
| 80 | 60 | 60 | partly cloudy | hot | strong | 66.67 |
| 80 | 70 | 100 | partly cloudy | warm | calm | 83.33 |
