# Supplementary material for: Dynamic multi-species occupancy models reveal individualistic habitat preferences in a high-altitude grassland bird community
Source: PeerJ. 2019 Feb 15;7:e6276. doi: 10.7717/peerj.6276 (PMC6378914; doi:10.7717/peerj.6276)
Supplement: Appendix S2 [file peerj-07-6276-s002.docx]

################################################################################################

R script with the JAGS model specification for multi-species hierarchical occupancy model with effect of grass height and grass cover on occupancy (Ψ), persistence (φ) and colonisation (γ) probabilities with additional effect of environment (cloud cover and wind) on detection probability (p).

#### **Packages to install**

library(lattice) #

library(gridExtra)

library(coda)

library(R2WinBUGS)

bugs.dir <- "C:\\Program Files\\WinBUGS14"

#install.packages(rjags)

library(rjags)

library(R2jags)

source("functions.r")

source("bugsmodels_2018_09_14.r")

###### **Bird data**

Allplots_birds_veg <- read.csv ("**Birds_veg_plot data seasons all rvsn.csv**")

Allplots_2012 <- Allplots_birds_veg[Allplots_birds_veg$Season=="Summer 2011-12",] # just analysing summer 2011-12

Allplots_2012$Plot<-factor(Allplots_2012$Plot) # drops unused levels of "Plot"

summary(Allplots_2012)

# Look at data

head(Allplots_2012)

str(Allplots_2012)

Allplots_2012$Surveyno <- ifelse(Allplots_2012$Month=="Dec", Allplots_2012$Survey + 3, ifelse(Allplots_2012$Month== "Jan", Allplots_2012$Survey+6, ifelse(Allplots_2012$Month =="Feb", Allplots_2012$Survey+9, Allplots_2012$Survey)))

Allplots_2012$Surveynof <- as.factor(paste(Allplots_2012$Month, Allplots_2012$Survey))

Allplots_2012$Date <- as.Date(Allplots_2012$Date)

Allplots_2012$day <- as.numeric(Allplots_2012$Date - min(Allplots_2012$Date))

##### **Vegtn file containing vegetation survey on which birds were recorded earlier**

#vegbirds <- read.csv("**Study plots vegetation 2010-12.csv**")

# Vegetation data

veg <- read.csv("Study plots vegetation 2010-12.csv") # vegetation survey file

veg$avh <- (veg$H1 + veg$H2 + veg$H3 + veg$H4)/4

veg$monthnumber <- ifelse(veg$Month=="Nov", 1, ifelse(veg$Month=="Dec", 2, ifelse(veg$Month=="Jan", 3, 4)))

veg.11 <- veg[veg$Survey=="Summer 11 - 12",]

#### **Insect data survey was not used in this analysis**

Insects <- read.csv ("Ingula insects survey 2011-12.csv") # insects survey file

Insects$monthnumber <- ifelse(Insects$Month=="Nov", 1, ifelse(Insects$Month=="Dec", 2, ifelse(Insects$Month=="Jan", 3, 4)))

# prepare covariates (will be the same for all species)

plots <- data.frame(as.vector(levels(Allplots_2012$Plot)))

unique(plots)

# site level covariates

where <- prepareSiteCovs(plots, Allplots_2012[,c("Plot", "Where")]) #where plot was at Ingula or neighbouring farms

unique(where)

# reviewers requires us to include the effect of observability (obs covs)& time (obsday) on detection

# observation level covariates

observibility <- prepareObsCovs(plots, Allplots_2012[,c("Plot","Surveyno","X2nd.Obserbility")])

colnames(observibility) <- c("plot", "obs.1", "obs.2", "obs.3", "obs.4", "obs.5", "obs.6", "obs.7", "obs.8", "obs.9", "obs.10", "obs.11", "obs.12")

obsday <- prepareObsCovs(plots, Allplots_2012[,c("Plot","Surveyno","day")])

colnames(obsday) <- c("plot", "day.1", "day.2", "day.3", "day.4", "day.5", "day.6", "day.7", "day.8", "day.9", "day.10", "day.11", "day.12")

# "yearly-site" level covariates

insects <- prepareObsCovs(plots, Insects[,c("Plot", "monthnumber", "wetweight")], fun=mean) # was not used in this analysis

avh <- prepareObsCovs(plots, veg.11[,c("Plot", "monthnumber", "avh")], fun=mean)

cover <- prepareObsCovs(plots, veg[,c("Plot", "monthnumber", "Grass")], fun=mean)

# prepare the data for WinBUGS - multi species models

# ****************************************************

sort(unique(Allplots_2012$Species))

# !! correcting errors and inconsistencies in data file!!

#Allplots_2012$Species[Allplots_2012$Species=="Cisticola Wing-snapping"] <- "Cisticola Wing-snapping"

#Allplots_2012$Species[Allplots_2012$Species=="Cisticola zitting"] <- "Cisticola Zitting"

# Selecting species that were used in this article

specieslist <- c("Pipit African", "Longclaw Cape", "Cisticola Wing-snapping", "Lark Red-capped", "Cisticola Zitting",

"Pipit Yellow-breasted", "Quail Common", "Widowbird Long-tailed", "Quailfinch African", "Martin Banded",

"Chat Ant-eating", "Lark Eastern Long-billed")

seasons <- 4

maxrep <- 3

nplots <- 19

nspecies <- length(specieslist)

y <- array(dim=c(nplots, maxrep, seasons, nspecies))

for (s in 1:nspecies) {

y.t <- selectSpecies(species=specieslist[s], seasons=seasons, maxrep=maxrep, nplots=nplots)

y[,,,s] <- y.t

}

str(y)

# covariates:

# **sobs & sobsday will make graphs for detection**

sobs <- scale(as.numeric(as.matrix((observibility[,2:dim(observibility)[2]]))))

sobs[is.na(sobs)] <- 0

obs <- array(dim=c(nplots, maxrep, seasons))

for (i in 1:seasons) obs[,,i] <- as.matrix(sobs[(1+(nplots*maxrep*(i-1))):(nplots*maxrep*i)])

sobsday <- scale(as.numeric(as.matrix((obsday[,2:dim(obsday)[2]]))))

sobsday[is.na(sobsday)] <- 0

oday <- array(dim=c(nplots, maxrep, seasons))

for (i in 1:seasons) oday[,,i] <- as.matrix(sobsday[(1+(nplots*maxrep*(i-1))):(nplots*maxrep*i)])

# scaling covariates

savh <- scale(as.numeric(as.matrix(avh[,2:dim(avh)[2]])))

savh[is.na(savh)] <- 0

davh <- matrix(as.numeric(savh), ncol= dim(y)[3])

scover <- scale(as.numeric(as.matrix(cover[,2:dim(cover)[2]])))

scover[is.na(scover)] <- 0

dcover <- matrix(as.numeric(scover), ncol= dim(y)[3])

# ************************************************************************************

# Model with species-random effects on detection and covariates on detection and state

# Bundle data

win.data <- list(y = y, nsite = dim(y)[1], nrep = dim(y)[2], nyear = dim(y)[3], nspecies = dim(y)[4], obs=obs, oday = oday, avh=davh, cover=dcover)

# Initial values

Zst <- array(dim=c(nplots, seasons, nspecies))

for (s in 1:nspecies) Zst[,,s] <- apply(y[,,,s], c(1, 3), max) # Observed occurrence as inits for z

inits <- function(){ list(z = Zst, mpsi = runif(1, -1, 1),

mphi = runif(1,-1, 1),

mgam = runif(1,-1, 1),

bpsiavh = runif(1,-1, 1),

bphiavh = runif(1,-1, 1),

bgamavh = runif(1,-1, 1),

bphicov = runif(1,-1, 1),

bpsicov = runif(1,-1, 1),

bgamcov = runif(1,-1, 1),

betaobs.mean = runif(1,-1, 1),

betaday.mean = runif(1,-1, 1),

tau.psi = runif(1,0,3),

tau.phi = runif(1,0,3),

tau.gam = runif(1,0,3),

tau.bpsiavh = runif(1,0,3),

tau.bphiavh = runif(1,0,3),

tau.bgamavh = runif(1,0,3),

tau.bphicov = runif(1,0,3),

tau.bpsicov = runif(1,0,3),

tau.bgamcov = runif(1,0,3),

tau.betaobs = runif(1,0,3),

tau.betaday = runif(1,0,3),

tau.p.mean = runif(1,0,3))}

# sig.p will replace mu.p to make detection graphs

# Parameters monitored

params <- c("n.occ", "srichness", "siterichness", "mu.p.mean", "sig.p.mean", "mu.p",

"mu.psi", "mu.phi","mu.gam","betapsiavh","betaphiavh","betagamavh","betaphicov","betapsicov", "betagamcov",

"mpsi","mphi","mgam","bpsiavh","bphiavh","bgamavh","bphicov","bpsicov","bgamcov","betaobs","betaobs.mean","betaday","betaday.mean",

"sig.psi","sig.phi","sig.gam","sig.bpsiavh","sig.bphiavh","sig.bgamavh","sig.bphicov","sig.bpsicov","sig.bgamcov",

"sig.betaobs","sig.betaday")

# MCMC settings

ni <- 60000

nt <- 20

nb <- 30000

nc <- 3

#############################################################################################

# Call JAGS from R ! runs twice as fast as WinBUGS !

start <- Sys.time()

# Compile the model.

N.mod <- jags.model("MultiSpeciesDynocc_2018_08_28.txt", win.data, inits, n.chains=3, n.adapt=nb)

# start time 14:25 - 14:53

# Draw samples from the posterior

# unused parameters in above .txt file

# start time 14:53 - 15:23

Nc <- coda.samples(N.mod, params, n.iter=ni-nb)

end <- Sys.time()

print(difftime(end, start, unit = "hours"),3)

# View the Markov chains

op <- par(mar=par("mar")/2)

# plot density trace plots

plot(Nc, ask=F)

#

sum.Nc <- summary(Nc)

par (op)

# Test for convergence

gelman.diag(Nc)

g <- matrix(NA, nrow=nvar(Nc), ncol=2)

for (v in 1:nvar(Nc)) {

g[v,] <- gelman.diag(Nc[,v])$psrf

}

g

save(sum.Nc, Nc, file="MultiSpeciesDynoccRandCovs3.RData")

load("MultiSpeciesDynoccRandCovs3.RData")

#######################################################################################

#######################################################################################

# plot influence of observibility covariates on detection

# replace avh with orbs covs and mpsi with p

**# PeerJ prefers pdfs**

pdf("**Fig 2** Effect of observability (sobs) and day of survey (betaday) covariate on detection of 12 spcs.pdf",width=11,height=6.5)

op<-par(mfrow=c(1,2), mar=c(5,5,1,1))

# Fig 4 - this code plots the mean, bold black line

# choose colours to represent each species

mycolours<-1:12 # c("azure",)

mycolours<- c("blue", "orange", "pink", "green", "purple", "red", "brown", "yellow", "olivedrab", "black", "azure4", "grey")

# effect of oservability covariates

x <- seq(min(sobs),max(sobs),0.1)

plot(x*attr(sobs,"scaled:scale")+attr(sobs,"scaled:center"), expit(sum.Nc$quantiles["mu.p.mean",3] + sum.Nc$quantiles["betaobs.mean",3] * x),

type='l', las=1, lwd=2, ylab="Detection probability", xlab="observability", ylim=c(0,1))

# this line below plots the species curves col=i produces better colours than col=rainbow(12)[i]

for (i in 1:nspecies) lines(x=x*attr(sobs,"scaled:scale")+attr(sobs,"scaled:center"), col = mycolours[i], y=expit(sum.Nc$quantiles[paste("mu.p[",i,"]", sep=""),3] + sum.Nc$quantiles[paste("betaobs[",i,"]", sep=""),3] * x))

#find out for which species, the model predicts either 0 or 1. - avh

# i = 6

# plot(ylim = c(0,1), x*attr(savh,"scaled:scale")+attr(savh,"scaled:center"), expit(sum.Nc$quantiles[paste("mu.psi[",i,"]", sep=""),3] + sum.Nc$quantiles[paste("betapsiavh[",i,"]", sep=""),3] * x), ylab = c("prob"))

# effect of day on detection

x <- seq(min(sobsday),max(sobsday),0.1)

plot(x*attr(sobsday,"scaled:scale")+attr(sobsday,"scaled:center"), expit(sum.Nc$quantiles["mu.p.mean",3] + sum.Nc$quantiles["betaday.mean",3] * x),

type='l', las=1, lwd=2, ylab="Detection probability", xlab="Day of survey", ylim=c(0,1))

# this line below plots the species curves col=i produces better colours than col=rainbow(12)[i]

for (i in 1:nspecies) lines(x=x*attr(sobsday,"scaled:scale")+attr(sobsday,"scaled:center"), col = mycolours[i], y=expit(sum.Nc$quantiles[paste("mu.p[",i,"]", sep=""),3] + sum.Nc$quantiles[paste("betaday[",i,"]", sep=""),3] * x))

# Species abreviations

# shortnames<- c("AP", "CLc", "WsC", "RcL", "ZC", "YbP", "CQ", "LtW", "AQf", "BMtn", "AEC", "ELL")

# legend(0,1,legend=shortnames[1:4],col=mycolours[1:4],lty=1,bty="n")

# legend(12,1,legend=shortnames[5:8],col=mycolours[5:8],lty=1,bty="n")

# legend(24,1,legend=shortnames[9:12],col=mycolours[9:12],lty=1,bty="n")

par(op)

dev.off()

####################################################################################

# Number of species occupying each plot (19 plots)

pdf("**Fig 3** Number of plots occupied for each species20181115_PeerJFNL.pdf",width=15,height=6.5)

#par(oma=c(3,3,0,0),mar=c(3,3,2,2),mfrow=c(2,2))

op <- par(mfrow=c(3,2), mar=c(4,0,1,0), oma=c(3,3,0,0))

index.n.occ <- list(c(157:160), c(161:164), c(165:168), c(169:172), c(173:176), c(177:180),

c(181:184), c(185:188), c(189:192), c(193:196), c(197:200), c(201:204))

speciesnames=c("African Pipit", "Cape Longclaw", "Wing-snapping Cisticola", "Red-capped Lark", "Zitting Cisticola",

"Yellow-breasted Pipit", "Common Quail", "Long-tailed Widowbird", "African Quailfinch", "Banded Martin",

"Ant-eating Chat", "Eastern Long-billed Lark")

plotoccupancy.jags(out=sum.Nc, K=seasons, nspecies = nspecies, specieslist=specieslist,

speciesnames=speciesnames, index.n.occ=index.n.occ)

par (op)

dev.off()

# Quantifying change in occupancy between Nov and Feb (as requested by one of the PeerJ reviewers)

#**********************************************************************************************

# the code below calculates the difference in the number of plots that were occupied in Nov and those that were occupied in February

# a positive number means that more plots were occupied in November

# if the credible interval includes 0, we don't have strong evidence for a change

for (i in 1:length(speciesnames)){

print(speciesnames[i])

print(quantile(rbind(Nc[[1]][,index.n.occ[[i]][1]], Nc[[2]][,index.n.occ[[i]][1]], Nc[[3]][,index.n.occ[[i]][1]]) - rbind(Nc[[1]][,index.n.occ[[i]][4]], Nc[[2]][,index.n.occ[[i]][4]], Nc[[3]][,index.n.occ[[i]][4]]), probs=c(0.025, 0.5, 0.975))) # 2.5th, 50th and 97.5th percentile -> credible interval for difference

}

# Eastern Long-billed Lark decline between January and February

print(quantile(rbind(Nc[[1]][,203], Nc[[2]][,203], Nc[[3]][,203]) - rbind(Nc[[1]][,204], Nc[[2]][,204], Nc[[3]][,204]), probs=c(0.025, 0.5, 0.975))) # 2.5th, 50th and 97.5th percentile -> credible interval for difference

# #################################################################################################

pdf("Fig 4 occupancy-persistence-colonization of 12 spcs20181115 PeerJ_Fnl.pdf",width=11,height=6.5)

op<-par(mfrow=c(2,3), mar=c(5,5,1,1))

# Fig 4 - this code plots the mean, bold

#colors() list of colours to choose from, we chose same colours those in Fig. 3

#cl<-colors()

#length(c);cl[1:50]

mycolours<-1:12 # c("azure",)

#mycolours<- c("brown", "pink4", "green", "magenta", "olivedrab4", "red", "paleturquoise4", "purple", "grey", "black", "blue", "orange")

mycolours<- c("blue", "orange", "pink", "green", "purple", "red", "brown", "yellow", "olivedrab", "black", "azure4", "grey")

x <- seq(min(savh),max(savh),0.1)

plot(x*attr(savh,"scaled:scale")+attr(savh,"scaled:center"), expit(sum.Nc$quantiles["mpsi",2] + sum.Nc$quantiles["bpsiavh",3] * x),

type='l', las=1, lwd=2, ylab="Occupancy probability", xlab="Grass height", ylim=c(0,1))

# this line below plots the species curves col=i produces better colours than col=rainbow(12)[i]

for (i in 1:nspecies) lines(x*attr(savh,"scaled:scale")+attr(savh,"scaled:center"), col = mycolours[i], expit(sum.Nc$quantiles[paste("mu.psi[",i,"]", sep=""),3] + sum.Nc$quantiles[paste("betapsiavh[",i,"]", sep=""),3] * x))

#find out for which species, the model predicts either 0 or 1. - avh

# i = 6

# plot(ylim = c(0,1), x*attr(savh,"scaled:scale")+attr(savh,"scaled:center"), expit(sum.Nc$quantiles[paste("mu.psi[",i,"]", sep=""),3] + sum.Nc$quantiles[paste("betapsiavh[",i,"]", sep=""),3] * x), ylab = c("prob"))

plot(x*attr(savh,"scaled:scale")+attr(savh,"scaled:center"), expit(sum.Nc$quantiles["mphi",2] + sum.Nc$quantiles["bphiavh",3] * x),

type='l', lwd=2, las=1, ylab="Persistence probability", xlab="Grass height", ylim=c(0,1))

for (i in 1:nspecies) lines(x*attr(savh,"scaled:scale")+attr(savh,"scaled:center"),col = mycolours[i], expit(sum.Nc$quantiles[paste("mu.phi[",i,"]", sep=""),3] + sum.Nc$quantiles[paste("betaphiavh[",i,"]", sep=""),3] * x))

#find out for which species, the model predicts either 0 or 1. - avh

#i = 4

#plot(ylim = c(0,1), x*attr(savh,"scaled:scale")+attr(savh,"scaled:center"), expit(sum.Nc$quantiles[paste("mu.phi[",i,"]", sep=""),3] + sum.Nc$quantiles[paste("betaphiavh[",i,"]", sep=""),3] * x), ylab = c("prob"))

plot(x*attr(savh,"scaled:scale")+attr(savh,"scaled:center"), expit(sum.Nc$quantiles["mgam",3] + sum.Nc$quantiles["bgamavh",3] * x),

type='l', lwd=2, las=1, ylab="Colonisation probability", xlab="Grass height", ylim=c(0,1))

for (i in 1:nspecies) lines(x*attr(savh,"scaled:scale")+attr(savh,"scaled:center"),col = mycolours[i], expit(sum.Nc$quantiles[paste("mu.gam[",i,"]", sep=""),3] + sum.Nc$quantiles[paste("betagamavh[",i,"]", sep=""),3] * x))

#find out for which species, the model predicts either 0 or 1. - avh

#i = 12

#plot(ylim = c(0,1), x*attr(savh,"scaled:scale")+attr(savh,"scaled:center"), expit(sum.Nc$quantiles[paste("mu.gam[",i,"]", sep=""),3] + sum.Nc$quantiles[paste("betagamavh[",i,"]", sep=""),3] * x), ylab = c("prob"))

shortnames<- c("AP", "CLc", "WsC", "RcL", "ZC", "YbP", "CQ", "LtW", "AQf", "BMtn", "AEC", "ELL")

legend(0,1,legend=shortnames[1:4],col=mycolours[1:4],lty=1,bty="n")

legend(12,1,legend=shortnames[5:8],col=mycolours[5:8],lty=1,bty="n")

legend(24,1,legend=shortnames[9:12],col=mycolours[9:12],lty=1,bty="n")

# grass cover

x <- seq(min(scover),max(scover),0.1)

plot(x*attr(scover,"scaled:scale")+attr(scover,"scaled:center"), expit(sum.Nc$quantiles["mpsi",3] + sum.Nc$quantiles["bpsicov",3] * x),

type='l', las=1, lwd=2, ylab="Occupancy probability", xlab="Grass cover", ylim=c(0,1))

for (i in 1:nspecies) lines(x*attr(scover,"scaled:scale")+attr(scover,"scaled:center"),col = mycolours[i], expit(sum.Nc$quantiles[paste("mu.psi[",i,"]", sep=""),3] + sum.Nc$quantiles[paste("betapsicov[",i,"]", sep=""),3] * x))

#find out for which species, the model predicts either 0 or 1. - cover

#i = 12

#plot(ylim = c(0,1), x*attr(scover,"scaled:scale")+attr(scover,"scaled:center"), expit(sum.Nc$quantiles[paste("mu.psi[",i,"]", sep=""),3] + sum.Nc$quantiles[paste("betapsicov[",i,"]", sep=""),3] * x))

plot(x*attr(scover,"scaled:scale")+attr(scover,"scaled:center"), expit(sum.Nc$quantiles["mphi",3] + sum.Nc$quantiles["bphicov",3] * x),

type='l', lwd=2, las=1, ylab="Persistence probability", xlab="Grass cover", ylim=c(0,1))

for (i in 1:nspecies) lines(x*attr(scover,"scaled:scale")+attr(scover,"scaled:center"),col = mycolours[i], expit(sum.Nc$quantiles[paste("mu.phi[",i,"]", sep=""),3] + sum.Nc$quantiles[paste("betaphicov[",i,"]", sep=""),3] * x))

#find out for which species, the model predicts either 0 or 1. - cover

#i = 1

#plot(ylim = c(0,1), x*attr(scover,"scaled:scale")+attr(scover,"scaled:center"), expit(sum.Nc$quantiles[paste("mu.phi[",i,"]", sep=""),3] + sum.Nc$quantiles[paste("betaphicov[",i,"]", sep=""),3] * x))

# centering and scaling grass cover

plot(x*attr(scover,"scaled:scale")+attr(scover,"scaled:center"), expit(sum.Nc$quantiles["mgam",3] + sum.Nc$quantiles["bgamcov",3] * x),

type='l', lwd=2, las=1, ylab="Colonisation probability", xlab="Grass cover", ylim=c(0,1))

for (i in 1:nspecies) lines(x*attr(scover,"scaled:scale")+attr(scover,"scaled:center"),col = mycolours[i], expit(sum.Nc$quantiles[paste("mu.gam[",i,"]", sep=""),3] + sum.Nc$quantiles[paste("betagamcov[",i,"]", sep=""),3] * x))

#find out for which species, the model predicts either 0 or 1. - cover

#i = 1

#plot(ylim = c(0,1), x*attr(scover,"scaled:scale")+attr(scover,"scaled:center"), expit(sum.Nc$quantiles[paste("mu.gam[",i,"]", sep=""),3] + sum.Nc$quantiles[paste("betagamcov[",i,"]", sep=""),3] * x))

par(op)

dev.off()

###################### **comparing bird species richness on Eskom farms with neighbours farms** ######################

# we first need to specify which rows we need

rownames(sum.Nc$quantiles) # to see the rownames

#rowsnov <-c(196,200,204,208,212,216,220,224,228,232,236,240,244,248,252,256,260,264,268)

# must be 19

rowsnov <-c(236,240,244,248,252,256,260,264,268,272,276,280,284,288,292,296,300,304,308)

srichnessnov<-sum.Nc$quantiles[rowsnov,3]

# 19 plots in nov

rowsdec <-c(237,241,245,249,253,257,261,265,269,273,277,281,285,289,293,297,301,305,309)

srichnessdec<-sum.Nc$quantiles[rowsdec,3]

rowsjan <-c(238,242,246,250,254,258,262,266,270,274,278,282,286,290,294,298,302,306,310)

srichnessjan<-sum.Nc$quantiles[rowsjan,3]

rowsfeb <-c(239,243,247,251,255,259,263,267,271,275,279,283,287,291,295,299,303,307,311)

srichnessfeb<-sum.Nc$quantiles[rowsfeb,3]

#op <- par(mfrow=c(2,2), par("mar"=c(2,3,3,0)), oma=c(2,1,0,1))

pdf("**Fig 5** Plot species richness, Eskom vs Neighbouring farms20181115_PeerJFNL.pdf",width=8,height=8)

op <- par(mfrow=c(2,2), (mar=c(3,3,1,1)), oma=c(0,3,0,0))

# November

boxplot(srichnessnov~where$Where, ylim=c(0,12), main="November", axes=F)

axis(side=2)

# December

boxplot(srichnessdec~where$Where, ylim=c(0,12), main="December", axes=F)

axis(side=2)

# January

boxplot(srichnessjan~where$Where, ylim=c(0,12), main="January", frame="F")

#axis(side=2)

#axis(side=1)

# February

boxplot(srichnessfeb~where$Where, ylim=c(0,12), main="February", frame="F")

mtext(side = 2, "Species richness", line = 1, outer=T)

par (op)

dev.off()

# quantify difference in species richness between Eskom and Private, as requested by reviewer

# ***************************************************************************************

# November

sr_Eskom <- rbind(Nc[[1]][,rowsnov[where$Where=="Eskom"]], Nc[[2]][,rowsnov[where$Where=="Eskom"]], Nc[[3]][,rowsnov[where$Where=="Eskom"]]) # posterior for species richness on Eskom plots (rowbinding the 3 chains)

sr_Private <- rbind(Nc[[1]][,rowsnov[where$Where=="Private"]], Nc[[2]][,rowsnov[where$Where=="Private"]], Nc[[3]][,rowsnov[where$Where=="Private"]]) # posterior for species richness on Private plots (rowbinding the 3 chains)

quantile(apply(sr_Eskom,1,mean) - apply(sr_Private,1,mean), probs=c(0.025, 0.5, 0.975)) # 2.5th, 50th and 97.5th percentile -> credible interval for difference

# December

sr_Eskom <- rbind(Nc[[1]][,rowsdec[where$Where=="Eskom"]], Nc[[2]][,rowsdec[where$Where=="Eskom"]], Nc[[3]][,rowsdec[where$Where=="Eskom"]]) # posterior for species richness on Eskom plots (rowbinding the 3 chains)

sr_Private <- rbind(Nc[[1]][,rowsdec[where$Where=="Private"]], Nc[[2]][,rowsdec[where$Where=="Private"]], Nc[[3]][,rowsdec[where$Where=="Private"]]) # posterior for species richness on Private plots (rowbinding the 3 chains)

quantile(apply(sr_Eskom,1,mean) - apply(sr_Private,1,mean), probs=c(0.025, 0.5, 0.975)) # 2.5th, 50th and 97.5th percentile -> credible interval for difference

# January

sr_Eskom <- rbind(Nc[[1]][,rowsjan[where$Where=="Eskom"]], Nc[[2]][,rowsjan[where$Where=="Eskom"]], Nc[[3]][,rowsjan[where$Where=="Eskom"]]) # posterior for species richness on Eskom plots (rowbinding the 3 chains)

sr_Private <- rbind(Nc[[1]][,rowsjan[where$Where=="Private"]], Nc[[2]][,rowsjan[where$Where=="Private"]], Nc[[3]][,rowsjan[where$Where=="Private"]]) # posterior for species richness on Private plots (rowbinding the 3 chains)

quantile(apply(sr_Eskom,1,mean) - apply(sr_Private,1,mean), probs=c(0.025, 0.5, 0.975)) # 2.5th, 50th and 97.5th percentile -> credible interval for difference

# February

sr_Eskom <- rbind(Nc[[1]][,rowsfeb[where$Where=="Eskom"]], Nc[[2]][,rowsfeb[where$Where=="Eskom"]], Nc[[3]][,rowsfeb[where$Where=="Eskom"]]) # posterior for species richness on Eskom plots (rowbinding the 3 chains)

sr_Private <- rbind(Nc[[1]][,rowsfeb[where$Where=="Private"]], Nc[[2]][,rowsfeb[where$Where=="Private"]], Nc[[3]][,rowsfeb[where$Where=="Private"]]) # posterior for species richness on Private plots (rowbinding the 3 chains)

quantile(apply(sr_Eskom,1,mean) - apply(sr_Private,1,mean), probs=c(0.025, 0.5, 0.975)) # 2.5th, 50th and 97.5th percentile -> credible interval for difference

# 20181115 for PeerJ_FNL
